# The Inverse Problems for Computational Psychophysiology: Opinions and Insights

**DOI:** 10.34133/2022/9850248

**Published:** 2022-08-24

**Authors:** Bin Hu, Kun Qian, Ye Zhang, Jian Shen, Björn W. Schuller

**Affiliations:** ^1^School of Medical Technology, Beijing Institute of Technology, Beijing 100081, China; ^2^School of Mathematics and Statistics, Beijing Institute of Technology, Beijing 100081, China; ^3^GLAM–Group on Language, Audio, & Music, Imperial College London, UK; ^4^Chair of Embedded Intelligence for Health Care and Wellbeing, University of Augsburg, Germany

## 1. Introduction

Since a long time, measuring the psychological status of subjects in a quantitative paradigm is a challenging problem in the scientific community. It is known that there is not a direct way to measure the psychological quantities [1], whereas an emerging methodology, i.e., *computational psychophysiology* (CPP), was introduced [2]. The core idea of CPP is to explore the link between the psychological quantities and the physiological quantities, which the latter ones can be measured via ubiquitous equipment (e.g., a brain-computer interface device). Psychiatric diseases are usually accompanied by abnormal psychological status, which can be objectively quantified by psychophysiological quantities. Evaluating psychiatric diseases is of great significance for mental health. With the fast development of artificial intelligence, big data, wearables, and the internet of things, we can observe successful achievements in finding quantitative methods for evaluating the degree of psychiatric diseases (e.g., depression) under the guidance of CPP. Nevertheless, the underlying mechanisms of these engineering milestones are still “up in the air” [3].

Investigating the fundamentals of CPP is a prerequisite for strengthening our power to extend the knowledge frontiers of mental health and benefit from clinical practice. D. R. Bach et al. proposed the concept of the “psychophysiological inverse problem,” claiming that psychologists use the peripheral physiological quantities to infer psychological quantities [4]. In particular, compared to other domains (e.g., intelligent disease diagnosis), understanding the mechanism of the mind could even benefit the development of novel clinical treatment methods for psychiatric disease. Therefore, the inverse problem tool cannot only facilitate a more personalised and precised medicine but also help discover the inherited characteristics of the psychophysiology. It is reasonable to think that the fundamental mechanism of CPP can be validated and/or interpreted by introducing the methodology of mathematical inverse problems. By the language of mathematical inverse problems [5], the computational psychophysiological problems can be formulated through an abstract equation,

(1) $$\begin{array}{c} f\left(x\right)=y, \end{array}$$

where the mapping *f*, which is also called the forward model or psychophysiological model in the literature of CPP [4], represents the link between the psychological quantity of interest *x* (e.g., the degree of depression) and the measurable physiological quantity is given by *y* (e.g., the heart rate). As for most of inverse problems, the essential computational difficulty of inverse psychophysiological problems should be its mathematical *ill-posedness* (in the sense of Hadamard) [5]: (i) the uniqueness issue, i.e., for a measured physiological quantity *y*, there may exist several psychological quantities *x*, satisfying the established mathematical model (1); (ii) the stability issue, i.e., a tiny perturbation of the data (either as measurement error and/or as numerical approximation error) may give a large change in the solution. Mathematical techniques such as (Tikhonov) regularisation can be adopted to tackle such ill-posedness.

## 2. Methods

In the literature of CPP, two methodologies exist for quantitatively solving psychophysiological inverse problems:

- The methodology lent from data science (e.g., statistics and machine learning). This methodology is associated with the data-driven inverse problem, i.e., the regression problem. To be more precise, it is aiming to directly construct a “good” approximation (e.g., a neural network *𝒩*) of the inversion mapping *f*^−1^. Once the approximation of the inversion mapping *𝒩* has been constructed, it can be used to study patients' psychological quantity *x* by using the measured physiological data *y*, i.e., *x* ≈ *𝒩*(*y*)
- The methodology lent from the classical inverse problems of mathematical physics. This approach, which is based on the knowledge-driven inverse problem, consists of two steps. The first step is the mathematical modelling, which is aimed at building the (forward) mapping *f* according to a physical law (unlike the approaches from machine learning, the inversion mapping *f*^−1^ itself cannot be established by a physical law, since in most models, a psychological quantity can only be appearing as the parameters in the physics informed model. An intuitive interpolation is that the psychological quantity is usually considered as an antecedent cause of a physiological quantity, which can be measured through a given equipment.). The second step is to (numerically) solve the corresponding mathematical model (1) with an appropriate mathematical algorithm such as (Tikhonov) regularisation or Bayesian inversion

## 3. Discussion

For the above two methodologies of solving psychophysiological inverse problems, each has its advantages and disadvantages. Thanks to the development of supercomputers, the machine learning-based approaches demonstrate impressive results in the first stage of the quantitative study of psychophysiological inverse problems, where an accurate mathematical representation of the problem cannot be driven. This methodology is straightforward, and the theoretical guarantee is based on the *universal approximation theorem*. However, the shortcoming of machine learning-based approaches is threefold: (1) For obtaining a good result, it requires dense data in the sampling space, which may be very expensive and time consuming for many psychophysiological problems. (2) The developed mathematical model, e.g., a constructed neural network from the deep learning paradigm, has no physical meaning, which hinders the further investigation and deep understanding of an interesting phenomenon in psychophysiology. (3) Due to the mathematical ill-posedness of psychophysiological inverse problems, the true inversion mapping *f*^−1^ is usually not a continuous mapping, which means that a precise approximation of *f*^−1^ (e.g., by a neural network *𝒩*) does not offer an accurate prediction *y*.

In comparison with machine learning-based approaches, the knowledge-driven mathematical models have a solid foundation from natural sciences. Usually, they provide a good result but require a minimum degree of data. However, the establishment of an appropriate mathematical model (i.e., a meaningful formula of *f* in (1)) through a physical law is not an easy task. Indeed, in order to explore the hidden physical law that linked the psychological quantity of interest and the physiological quantity in a designed experiment, both deep domain knowledge (i.e., the knowledge of psychological or biophysical relationships) and tricky mathematical skills are required.

The authors in [4] offer a good review on statistical psychophysiological inverse problems. They focus on the (statistical) modelling of the corresponding forward and inverse problems, without any computational issues. Yet, they do not allude to how to build a forward model through a physical law. Moreover, they do not feature the mathematical ill-posedness of the psychophysiological inverse problems, which is the crucial computational difficulty in the numerical solution of the constructed model in practice. In the appendix of [4], they consider the maximum likelihood amplitude estimate (i.e., the conventional least square method), which is not a correct mathematical method for ill-posed inverse problems.

## 4. Outlook

To explore the inverse problems for CPP, we provide some perspectives and outlooks as follows:

First, we need to design novel paradigms for building the mathematical models between the psychological quantities and the physiological quantities. The inverse problem methodology can be a powerful tool to help us to find the solutions. Second, we need to collect multimodal behavioural and physiological data to explore the relationship between the psychological quantities and physiological measures from different views and eventually establish an objective and quantitative index system for psychological quantities. The inverse methodology is the fundamental theoretical basis for establishing the above index system.

Third, psychophysiological problems are commonly formulated in a static setting where a wealth of theoretical results and numerical algorithms are available. However, the real psychophysiological model is dynamical, where time-dependent information needs to be discerned from time-dependent data. Simplistic extension by indiscriminate inclusion of time as another dimension results in loss of information and changes the characteristics of the respective problem. Hence, dynamical psychophysiological problems will require the development of a new comprehensive framework, unfolding from modelling, through regularisation, to numerical methods.

Fourth, a real psychophysiological system always involves noise or uncertainty. This may be in the form of external disturbances as well as internal noise attributable to insufficient knowledge of the experiment or inaccuracy of the measuring systems. Hence, psychophysiological problems under uncertainties should be studied in the future. In particular, some stochastic psychophysiological models shall be investigated.

Fifth, since the psychophysiological inverse problems are usually mathematically ill-posed, some specific regularisation methods should be developed for accurate estimation of psychological quantity.

Last but not the least, recently, some physics-informed neural networks have been developed for solving some real-word problems. They can be viewed as hybrid methods combining both methods, i.e., those from data science and those from mathematical physics. Such methods require substantially less training data and can result in simpler neural network structures, while achieving high accuracy. Therefore, in our opinion, the physics-informed neural networks can be judged as a good candidate for modelling and efficient solving of psychophysiological inverse problems.
